# Novel real-time PCR-based patho- and phylotyping of potentially zoonotic avian influenza A subtype H5 viruses at risk of incursion into Europe in 2017

**DOI:** 10.2807/1560-7917.ES.2017.22.1.30435

**Published:** 2017-01-05

**Authors:** Mahmoud M Naguib, Annika Graaf, Andrea Fortin, Christine Luttermann, Ulrich Wernery, Nadim Amarin, Hussein A Hussein, Hesham Sultan, Basem Al Adhadh, Mohamed K Hassan, Martin Beer, Isabella Monne, Timm C Harder

**Affiliations:** 1 Institute of Diagnostic Virology, Friedrich Loeffler Institute, Greifswald-Riems, Germany; 2 National Laboratory for Veterinary Quality Control on Poultry Production, Animal Health Research Institute, Giza, Egypt; 3 Istituto Zooprofilattico Sperimentale delle Venezie, Padua, Italy; 4 Institute of Immunology, Friedrich Loeffler Institute, Greifswald-Riems, Germany; 5 Central Veterinary Research Laboratory (CVRL), Dubai, United Arab Emirates; 6 Boehringer Ingelheim, Dubai, United Arab Emirates; 7 Faculty of Veterinary Medicine, Cairo University, Giza, Egypt; 8 Birds and Rabbits Medicine Department, Faculty of Veterinary Medicine, Sadat City University, Egypt; 9 Central Veterinary Laboratory, Ministry of Agriculture, Baghdad, Iraq

**Keywords:** Zoonotic avian influenza, phylotyping, pathotyping, real-time RT-qPCR, diagnosis

## Abstract

Since November 2016, Europe witnesses another wave of incursion of highly pathogenic avian influenza (HPAI) A(H5) viruses of the Asian origin goose/Guangdong (gs/GD) lineage. Infections with H5 viruses of clade 2.3.4.4b affect wild bird and poultry populations. H5 viruses of clades 2.2, 2.3.1.2c and 2.3.4.4a were detected previously in Europe in 2006, 2010 and 2014. Clades 2.2.1.2 and 2.3.2.1.c are endemic in Egypt and Western Africa, respectively and have caused human fatalities. Evidence exists of their co-circulation in the Middle East. Subtype H5 viruses of low pathogenicity (LPAI) are endemic in migratory wild bird populations. They potentially mutate into highly pathogenic phenotypes following transmission into poultry holdings. However, to date only the gs/GD H5 lineage had an impact on human health. Rapid and specific diagnosis marks the cornerstone for control and eradication of HPAI virus incursions. We present the development and validation of five real-time RT-PCR assays (RT-qPCR) that allow sequencing-independent pathotype and clade-specific distinction of major gs/GD HPAI H5 virus clades and of Eurasian LPAI viruses currently circulating. Together with an influenza A virus-generic RT-qPCR, the assays significantly speed up time-to-diagnosis and reduce reaction times in a OneHealth approach of curbing the spread of gs/GD HPAI viruses.

## Introduction

Influenza A viruses constitute a virus species in the family *Orthomyxoviridae*. They harbour single-stranded negative-sense RNA arranged into eight genomic segments. Members of this species which infect avian hosts (avian influenza viruses, AIV) are grouped into 16 (H1 to H16) and 9 (N1 to N9) subtypes, respectively, based on phylogenetic and antigenic properties of their haemagglutinin (HA) and neuraminidase (NA) envelope glycoproteins [1]. Different species of aquatic wild birds are the natural reservoirs for all AIV subtypes. Novel subtypes and gene constellations continue to evolve in aquatic wild birds or in infected poultry populations by genetic reassortment during infection of a single host cell with two or more distinct AIV genotypes. In addition to reassortment, the intrinsically error-prone influenza virus genome replication machinery promotes the generation of quasi-species that can be shaped by directional selection pressures, e.g. following host species switches or by specific herd immunity. In the latter case, antigenic drift variants are selected that may escape immunity by very few amino acid substitutions in the HA [2].

Based on their virulence in galliform poultry (e.g. chicken, turkey), AIV are distinguished into groups of highly pathogenic (HP) and low pathogenic (LP) phenotypes [3]. Correct AI diagnosis includes determining the HA subtype and, in case of subtypes H5 or H7, also the pathotype. So far, HPAI phenotypes detected in the field (i.e. ‘free’ natural environment), were only described among AIV of subtypes H5 and H7 [4]. Some of these viruses including those of the HPAI H5 goose/Guangdong (gs/GD) lineage that emerged in southern China in 1996, have zoonotic potential and are sporadically transmitted from infected birds to humans [5,6]. HPAI viruses of the gs/GD lineage have continued to circulate and evolved into numerous clades. Viruses of three major phylogenetic clades (2.2.1.2, 2.3.2.1 and 2.3.4.4) as well as of three further minor clades (1.1.2, 2.1.3.2 and 7.2) have become endemic in poultry populations in several countries in Asia, Africa and the Middle East [7]. Occasionally, spillover transmission from infected poultry may cause infection and viral spread in wild birds with increased mortality in some species. Infected migratory wild birds may spread such viruses across wider distances and act as the source of transmission back to poultry [7,8].

Europe has experienced several incursions by viruses of the gs/GD lineage over the past decade; both wild birds and poultry were affected but no human cases were reported [9]. This is in sharp contrast to Egypt and Asian countries where the endemicity of HPAI H5 viruses in poultry is associated with repeated spillover transmission to and infection of humans. In fact, the majority of human HPAI H5 cases worldwide were registered in Egypt [10,11]. Moreover, a new major clade, designated 2.2.1.2, evolved along with transient spread of an escape mutant-based lineage, 2.2.1.1, in this country [12].

Further potentially zoonotic gs/GD viruses of clade 2.3.2.1c are widespread in Central and Southern Asia and they were sporadically detected along the European Black Sea coast as well as in the Middle East [13-15]. In addition, viruses of this clade have caused major outbreaks among poultry in several Western African countries with ongoing virus circulation to date [16]. Interestingly, 2.3.2.1c viruses have not (yet) been reported from Egypt. Since 2010, another gs/GD cluster, termed 2.3.4.4, has evolved in eastern China and on the Korean peninsula [17]. These viruses have revealed a strong propensity to reassort with other influenza subtypes giving rise to novel HPAI sub- and genotypes including influenza A(H5N6) and A(H5N8). The latter subtype has proven to be highly mobile and was carried by infected wild birds to Europe and the North American continent in late 2014 [8,18]. In November 2016, HPAI H5N8 viruses of the 2.3.4.4 clade re-emerged on a large scale in wild birds in several central European countries and caused considerable mortality especially among diving duck species; sporadic incursions into poultry holdings were documented as well [19]. At the same time, this lineage was also detected in poultry in Israel [20].

Eurasian-origin LPAI subtype H5 viruses distantly related to the gs/GD lineage are routinely detected in aquatic wild bird populations with peak incidences during the autumn migration period [21]. Spillover of LPAI virus into poultry may cause notifiable outbreaks and bears the risk of the de novo generation of HP phenotypes following spontaneous mutations [3]. No human LPAI H5 virus infections have been reported so far.

Continuous co-circulation in poultry and sporadic spillover into migratory wild bird populations of different endemic HPAI H5 virus lineages poses constant risks of new incursions into Europe by migrating wild birds or in association with (illegal) poultry trading practices [9]. Furthermore, co-circulation of various HPAI lineages with different antigenic properties potentiates problems of control and eradication. Given the zoonotic propensities of some of the H5 viruses, tight control of infections in poultry is essential to curtail risks of human infections and further spread [22,23]. Molecular diagnosis including patho- and phylotyping of the relevant AIV is an important prerequisite for effective control measures.

We developed rapid diagnostic solutions on the basis of quantitative reverse transcription real-time PCR assays (RT-qPCR), to pathotype, without sequencing, gs/GD lineage HPAI and Eurasian LPAI H5 subtype viruses, and to distinguish HPAI gs/GD viruses of clades 2.2.1.2, 2.3.2.1 and 2.3.4.4, including viruses of the ongoing 2016 epizootic in Europe.

## Methods

### Virus isolates and clinical samples

A total of 24 reference virus isolates were obtained from the virus repositories at the Friedrich Loeffler Institute, Greifswald-Riems, Germany, or were provided by the National Laboratory for quality control on poultry production in Giza, Egypt, and by the Central Veterinary Research Laboratory (CVRL) in Dubai, United Arab Emirates (see also first table under Results).

Moreover, 106 field samples were included. These were obtained from holdings of different poultry sectors and wild birds from countries in Western Europe (Germany), the Middle East (Egypt, Iraq, United Arab Emirates) and Western Africa (Burkina Faso, Cameroon, Ghana, Ivory Coast, Niger), for HPAI viruses in the period between 2013 and 2016. Samples consisted mainly of oropharyngeal and/or cloacal swabs and tissues samples (n = 70) or AIV isolated from such samples (n = 36) (see also second table under Results).

A subsection of the 106 clinical samples (n = 13) was provided as dried material on Whatman FTA card (Sigma Aldrich, Germany). Samples from Western African countries were exclusively assayed at the Istituto Zooprofilattico Sperimentale delle Venezie, Padua, Italy.

### Design of primers and probes

Primers were chosen based on alignments of the HA H5 gene of a selection of influenza A virus sequences submitted over the past 10 years to GenBank at the National Center for Biotechnology Information (NCBI) or to the EpiFlu database of the Global Initiative on Sharing Avian Influenza Data (GISAID). Selected sequences represented Eurasian LP viruses and HP isolates and clades of the gs/GD lineage that were detected in Europe, the Middle East and Western Africa during the past decade. Selection of primers to amplify a small fragment of the HA gene spanning the endoproteolytic cleavage site aimed at being broadly inclusive so as to target as many of the published LP Eurasian H5 HA sequences as possible and to distinguish them from HP viruses of the gs/GD lineage. The probes were placed directly onto the cleavage site in the attempt to specifically bind to sequences encoding either mono- or polybasic patterns that distinguish LP and HP pathotypes, respectively (Table 1).

**Table 1:** Primers and probes designed for differentiating pathotype and phylotype of Eurasian wild bird and goose/Guangdong origin potentially zoonotic avian influenza A subtype H5 viruses

Primer/Probe ID	Target	Sequence (5’ to 3’)	Location	Amplicon size	Accession number^a^
H5_HP_EA_F1	HPAI H5	CCTTGCDACTGGRCTCAG	984–1001	109	EPI647540
H5_HP_EA_F2		TCCTTGCAACAGGACTAAG	983–1001		
H5_HP_EA_probe		FAM- AAGAARAAARAGAGGACTRTTTGGAGCT-BHQ-1	1023–1050		
H5_HP_EA_R		GTCTACCATTCCYTGCCA	1092–1075		
					
H5LP-EA_F	LPAI H5	CCCAAATACGTGAAATCAGAT	955–975	133	EPI356413
H5LP1_EA_probe		FAM-CCAAATAGYCCTCTYGTYTCT-BHQ-1	1052–1072		
H5LP-EA_R		GCC ACC CTC CTT CTA TAA AG	1088–1069		
					
H5_2.2.1.2_Fw	Clade 2.2.1.2	CATTTTGAGAAAATTCAGATCATT	376–399	161	EPI573250
H5_2.2.1.2_probe		FAM-TCCATACCARGGAAGATCCTCCTTT-BHQ-1	451–474		
H5_2.2.1.2_Rev		GGTATGCATCGTTCTTTTTGG	537–517		
					
H5_2.3.2.1_F	Clade 2.3.2.1	GAGATTGGTACCAAAAATAGCC	669–690	146	EPI603577
H5_2.3.2.1_probe		FAM-ACGGGCAAAGTGGCAGGATAGATTTC-BHQ-1	707–732		
H5_2.3.2.1_R		CAATGAAATTTCCATTACTCTCG	815–793		
					
H5_2.3.4.4_F_A	Clade 2.3.4.4	ATACCAGGGAGCATCCTCA	484–502	114	EPI554605
H5_2.3.4.4_F_B		ATACCAGGGAACGCCCTCC	484–502		
H5_2.3.4.4_probe		FAM-TCGTTCTTTTTGATGAGCCATACCACA-BHQ-1	540–560		
H5_2.3.4.4_R_A		ATTATTGTAGCTTATCTTTATTGTC	598–574		
H5_2.3.4.4_R_B		ATTATTGTAGCTTATCTTTATTGTT	598–574		

gs/GD: goose/Guangdong; HA: haemagglutinin; ID: identity.

^a^ Accession number used to describe the position of the oligonucleotide along the HA gene. Sequences were obtained from GenBank at the National Center for Biotechnology Information (NCBI) or the EpiFlu database of the Global Initiative on Sharing Avian Influenza Data (GISAID).

At first, sets of primers and probes were designed to detect and discriminate between HP and LP biotypes, i.e. Eurasian H5 viruses encoding a monobasic or a polybasic HA cleavage site. In addition, four different sets of primers and probes were developed to differentiate between gs/GD clades 2.2.1.2, 2.3.2.1 and 2.3.4.4 (A and B). Pre-selected primers were then screened in silico for their specificity properties using Shannon entropy plots implemented in the Entropy One software (http://www.hiv.lanl.gov/content/sequence/-ENTROPY/entropy_one.html). Oligont (oligont) were selected so as to retain full specificity for the selected clade and to maximise entropy against all other clades. Basic physical properties of oligont were checked using the online web interface Oligo Calculator version 3.27. The finally chosen oligont are shown in Table 1. Detailed results of the in silico analyses are available on request from the authors.

### One-step quantitative reverse transcription PCR assays

All reactions were performed using the AgPath-ID One-Step RT-qPCR kit (Thermofisher, scientific, United States) as follows: Reverse transcription at 45 °C for 10 min, initial denaturation at 95 °C for 10 min, 40 cycles of PCR amplification at 95 °C for 30 s, 58 °C for 15 s, and 72 °C for 15 s in a 25 µl reaction mixture using 15 pmol of each forward and reverse primers and 5 pmol probe per reaction. For each parameter a separate reaction was used. Cycling was performed on a Biorad CFX96 Real-Time cycler (BioRad, Germany). Fluorescent signals were collected during the annealing phase, and the amplification data were analysed using Bio-Rad CFX Manager 3 software accessing automated fluorescence drift correction for baseline adjustment.

### Nucleotide sequencing and clade assignment

Patho- and phylotyping results obtained by newly developed RT-qPCRs were counter-checked by nt (nt) sequencing of the entire or parts of the HA gene of the respective isolates/clinical samples. Amplification of the HA gene was performed using primers published previously [24] and primers recommended in the European Union Diagnostic Manual for AI in a one-step RT-PCR [25]. In addition, amplificates of the HPAI H5 and LPAI H5 RT-qPCRs were used for sequencing purposes as well. Products were size-separated in agarose gels, excised and purified using the QIAquick Gel Extraction Kit (Qiagen, Hilden, Germany). Purified PCR products were used for cycle sequencing reactions (BigDye Terminator v1.1 Cycle Sequencing Kit, Applied Biosystems, California, United States) the products of which were purified using NucleoSEQ columns (Macherey-Nagel GmbH and Co, Düren, Germany) and sequenced on an ABI PRISM 3130 Genetic Analyzer (Life Technologies, Darmstadt, Germany).

For pathotyping, deduced amino acid sequences of the endoproteolytical cleavage site of the HA gene were inspected and compared with the molecular pathotyping database provided by OFFLU [26]. Assignment of nt sequences to the gs/GD HPAI H5 virus clade system was performed by use of clade prediction tool implemented in the Influenza Research Database [27].

## Results

### Analytical specificity of pathotyping and phylotyping quantitative reverse transcription PCR assays

The specificity of the assays was evaluated with viral RNA from representative influenza A subtype H5 viruses that had been phylotyped based on full-length HA nt sequence analysis (Table 2). Furthermore, non-H5 subtypes, i.e. H9N2 and H7N7, as well as non-influenza avian viruses i.e. avian infectious bronchitis virus (IBV) and Newcastle disease virus (NDV) were employed (Table 2), and none of them was detected by any of the specific PCRs.

**Table 2:** Reference viruses used to determine analytical specificity of five PCR assays to detect potentially zoonotic avian influenza subtype H5 viruses

Reference virus		Accession number of HA^a^	Patho- and Phylotype	PCR method ^b^				
				HPAI H5	LPAI H5	Clade 2.2.1.2	Clade 2.3.2.1	Clade 2.3.4.4
1	A/turkey/Turkey/1/2005 (H5N1)	KF042153	HP Clade 2.2	**Pos**	**Neg**	**Pos**	**Neg**	**Neg**
2	A/chicken/Egypt/0879-NLQP/R737/2008 (H5N1)	GQ184238	HP Clade 2.2.1.**1**	**Pos**	**Neg**	**Neg**	**Neg**	**Neg**
3	A/chicken/Egypt/NLQP7FL-AR747/ 2013 (H5N1)	EPI557170	HP Clade 2.2.1.2	**Pos**	**Neg**	**Pos**	**Neg**	**Neg**
4	A/duck/Egypt/AR236-A3NLQP/2015 (H5N1)	EPI573260	HP Clade 2.2.1.2	**Pos**	**Neg**	**Pos**	**Neg**	**Neg**
5	A/turkey/Egypt/AR238-SD177NLQP/2014 (H5N1)	EPI573268	HP Clade 2.2.1.2	**Pos**	**Neg**	**Pos**	**Neg**	**Neg**
6	A/peregrine falcon/Dubai/AR3430/2014 (H5N1)	EPI603553	HP Clade 2.3.2.1c	**Pos**	**Neg**	**Neg**	**Pos**	**Neg**
7	A/quail/Dubai/AR3445–2504.3/2014 (H5N1)	EPI603577	HP Clade 2.3.2.1c	**Pos**	**Neg**	**Neg**	**Pos**	**Neg**
8	A/duck/Bangladesh/D3-AR2111/2013 (H5N1)	SA^c^	HP Clade 2.3.2.1a	**Pos**	**Neg**	**Neg**	**Pos**	**Neg**
9	A/turkey/Germany/AR2485–86/2014 (H5N8)	EPI552746	HP Clade 2.3.4.4a	**Pos**	**Neg**	**Neg**	**Neg**	**Pos**
10	A/turkey/Germany-MV/AR2472/2014 (H5N8)	EPI544756	HP Clade 2.3.4.4a	**Pos**	**Neg**	**Neg**	**Neg**	**Pos**
11	A/tufted duck/Germany/AR8444/2016 (H5N8)	EPI859212	HP Clade 2.3.4.4b	**Pos**	**Neg**	**Neg**	**Neg**	**Pos**
12	A/chicken/Indonesia/R132/2004 (H5N1)	EPI354072	HP Clade 2.1.1	**Pos**	**Neg**	**Neg**	**Neg**	**Neg**
13	A/chicken/Indonesia/R134/2003 (H5N1)	AM183669	HP Clade 2.1.1	**Pos**	**Neg**	**Neg**	**Neg**	**Neg**
14	A/chicken/Indonesia/R60/2005 (H5N1)	AM183670	HP Clade 2.1.1	**Pos**	**Neg**	**Neg**	**Neg**	**Neg**
15	A/Vietnam/1194/2004 (H5N1)	GQ149236	HP Clade 1.1	**Pos**	**Neg**	**Neg**	**Neg**	**Neg**
16	A/chicken/GXLA/1204/2004 (H5N1)	AM183671	HP Clade 2.4	**Pos**	**Neg**	**Neg**	**Neg**	**Neg**
17	A/chicken/Vietnam/P41/2005 (H5N1)	AM183672	HP Clade 1.1	**Pos**	**Neg**	**Neg**	**Neg**	**Neg**
18	A/chicken/Vietnam/P78/2005 (H5N1)	AM183673	HP Clade 1.1	**Pos**	**Neg**	**Neg**	**Neg**	**Neg**
19	A/common teal/Germany/Wv1378–79/2003 (H5N2)	HF563058	LP	**Neg**	**Pos**	**Neg**	**Neg**	**Neg**
20	A/duck/Germany/R1789/2008 (H5N3)	CY107849	LP	**Neg**	**Pos**	**Neg**	**Neg**	**Neg**
21	A/turkey/Germany/AR915/2015 (H7N7)	SA^c^	H7N7	**Neg**	**Neg**	**Neg**	**Neg**	**Neg**
22	A/chicken/Egypt/AR754–14/2013 (H9N2)	EPI557457	H9N2	**Neg**	**Neg**	**Neg**	**Neg**	**Neg**
23	A/chicken/Sudan/AR251–15/2014 (IBV)	KX272465	IBV	**Neg**	**Neg**	**Neg**	**Neg**	**Neg**
24	A/chicken/Egypt/AR254–15/2014 (NDV)	SA^c^	NDV	**Neg**	**Neg**	**Neg**	**Neg**	**Neg**

Cq: cycle of quantification; HA: haemagglutinin; HP: highly pathogenic; HPAI: highly pathogenic avian influenza; IBV: infectious bronchitis virus; LP: low pathogenic; LPAI: low pathogenic avian influenza; NDV: Newcastle disease virus; Neg: negative; Pos: positive; RT-qPCR: quantitative reverse transcription PCR; SA: sequences available.

^a^ Sequences were obtained from GenBank at the National Center for Biotechnology Information (NCBI) or the EpiFlu database of the Global Initiative on Sharing Avian Influenza Data (GISAID).

^b^ Positive results: Cq value in similar range as with influenza A virus generic M RT-qPCR; negative results: Cq > 40.  ^c^ Sequenced in the frame of the current study; sequences available from the authors upon request.

In the initial evaluation of the specificity of the pathotyping RT-qPCR assays carried out using two reference viruses: HPAI A/chicken/Egypt/AR236/2015 (H5N1, clade 2.2.1.2) and LPAI A/turkey/Germany/R2025/2008 (H5N3), specific reactivity exclusively with the homo-pathotypic virus was evident. In a second step, assays were extended to the full range of 24 reference viruses yielding a similar sharp distinction between HP and LP cleavage sites (Table 2).

Primers and probes for phylotyping RT-qPCR assays distinguishing three clades of gs/GD origin HPAIV H5 were placed within the HA1-fragment of the HA gene. This region encodes the receptor binding unit and harbours a number of neutralisation-relevant epitopes that are targets of antigenic drift. Hence, the HA1 fragment harbours regions that are the least conserved within the influenza A virus genome. Primer selection aimed at the inclusion of as many as possible distinguishing nt that would define exclusivity at the five most 3’ positions while probes were placed so as to accommodate distinguishing nt in the centre of the oligont. In order not to compromise amplification efficacy, amplicon size was limited to 130 nt wherever possible given the above mentioned constraints for primers and probes. The finally chosen oligont are listed in Table 1 and provided specific detection exclusively of the homologous clade. No cross-reactivity among the other gs/GD clades examined was evident on basis of the used panel of reference viruses (Table 2). Also, no cross-reactivity was detected for any of the five assays against other influenza A viruses or other avian viral respiratory pathogens (Table 2).

### Validation of the analytical sensitivity, limit of detection and precision

**Figure 1:**
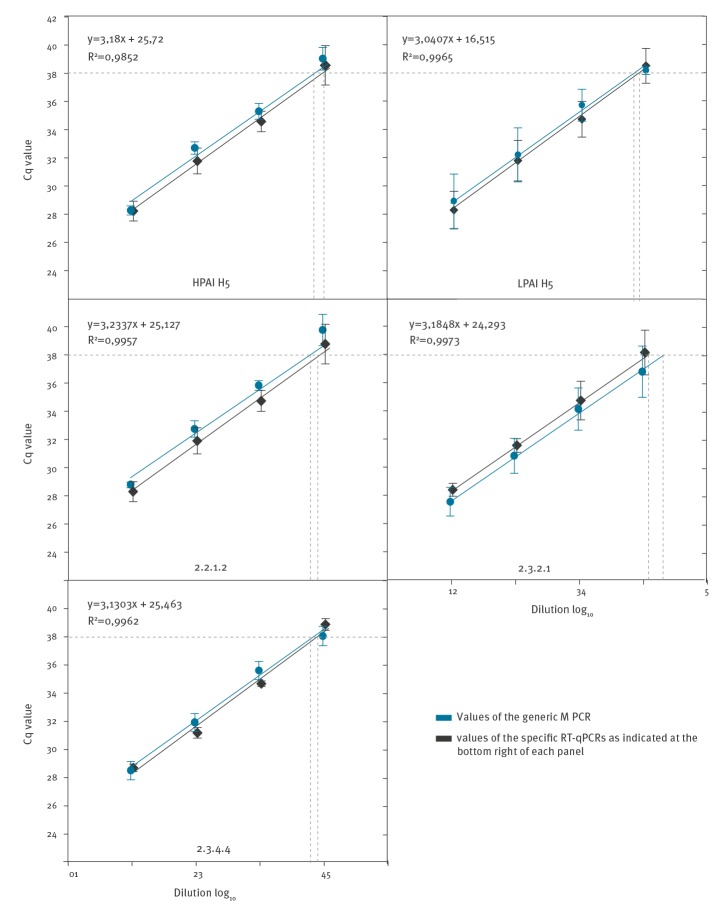
Evaluation of detection limits and precision of pathotyping and phylotyping quantitative reverse transcription PCRs compared with a generic matrix (M) gene RT-qPCR^a^

Detection limits of the assays were determined by testing 10-fold serial dilutions of viral RNA extracted from representative viruses of each of the three HPAI virus clades (2.2.1.2, 2.3.2.1 and 2.3.4.4), and of Eurasian H5 LPAI virus. Cycle of quantification (Cq) values were compared with a standard RT-qPCR for the matrix (M) gene of these viruses with a reported detection limit of 2 to 20 RNA copies/5 µl [28]. Average values of three separate runs were computed and plotted using SigmaPlot V 11 software. Plotting these values revealed a linear relationship between the log of the viral RNA dilution and the Cq value for all assays and the kinetics of the assays and their sensitivity were determined to be very similar to the generic M gene RT-qPCR (M1.2 RT-qPCR [29]) (Figure 1).

Cq: cycle of quantification; HPAI: highly pathogenic avian influenza; LPAI: low pathogenic avian influenza; M: matrix; RT-qPCR: quantitative reverse transcription PCR.

The detection limit was determined based on triplicate analyses of serial 10-fold dilutions of target RNA of reference viruses: HPAI H5: A/duck/Egypt/AR236-A3NLQP/2015 (H5N1); LPAI H5: A/teal-Foehr/Wv1378–79/2003 (H5N2) (upper panel); HPAI H5 clade 2.2.1.2: A/duck/Egypt/AR236-A3NLQP/2015 (H5N1); clade 2.3.2.1: A/quail/Dubai/AR3445–2504.3/2014 (H5N1) and clade 2.3.4.4: A/turkey/Germany-MV/R2472/2014 (H5N8) (lower panel). A cut-off value of Cq 38 was chosen to calculate limits of detection and confidence intervals thereof.

^a^ Described in [29].

The correlation coefficient of the standard curves was 0.99 for all assays, indicating a highly precise log–linear relationship between the viral RNA log dilution and the corresponding Cq-value (Figure 1). Based on these results the threshold distinguishing positive and negative was set at Cq = 38.

### Pathotyping and phylotyping of clinical samples of potentially zoonotic Eurasian avian influenza A subtype H5 viruses by quantitative reverse transcription PCR

In order to evaluate the diagnostic performance capacity of the developed assays, field samples (RNA extracted from swabs, tissues or FTA cards) and clinical virus isolates obtained during the period 2013 to 2016 (HPAI viruses) or 2003 to 2015 (LPAI viruses) were examined. The sample set was preselected on basis of a positive generic M-specific RT-qPCR.

**Figure 2:**
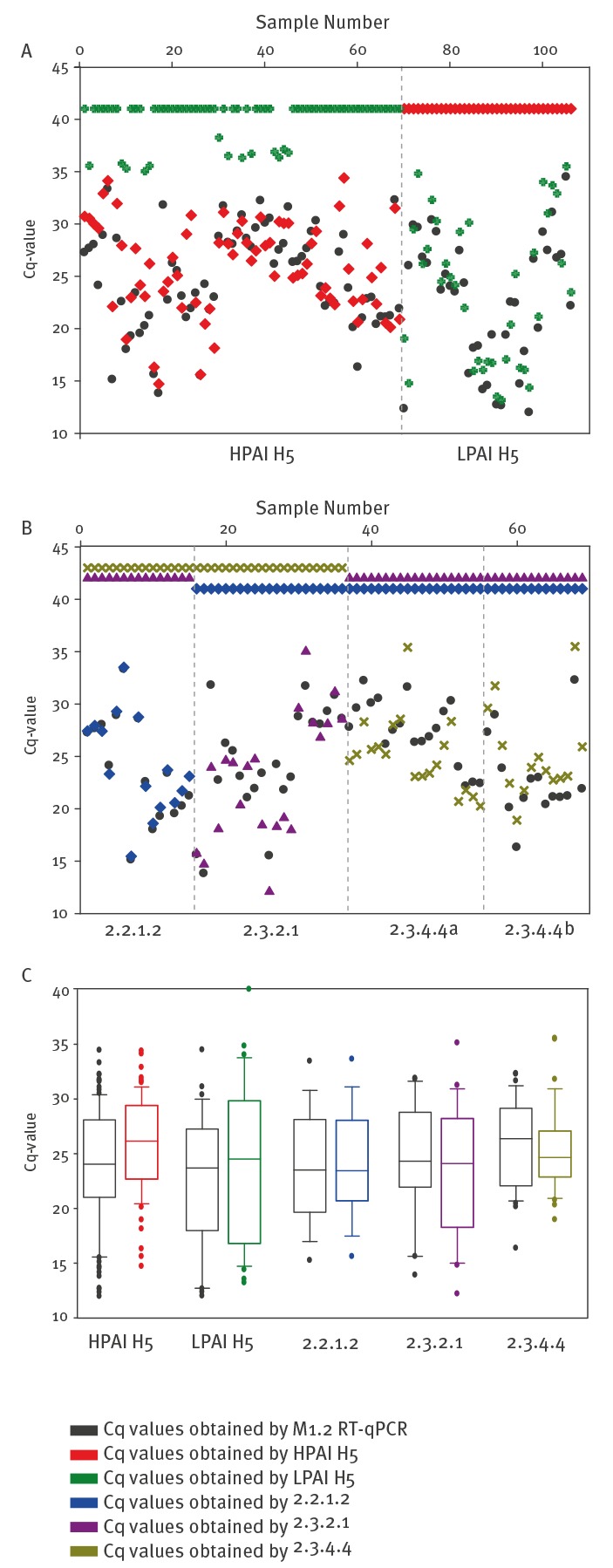
Pathotyping and phylotyping of virus isolates and clinical samples of potentially zoonotic Eurasian avian influenza A subtype H5 viruses by quantitative reverse transcription PCRs

Among the final set of 106 samples, the pathotyping RT-qPCRs sharply discerned two groups of 69 samples reacting only in the new HPAI H5 RT-qPCR while 37 samples reacted positive in the LPAI H5 RT-qPCR (Figure 2a; Table 3).

Cq: cycle of quantification; HPAI: highly pathogenic avian influenza; LPAI: low pathogenic avian influenza; M: matrix; RT-qPCR: quantitative reverse transcription PCR.

Sample numbers in A and B refer to Table 3. Cq values obtained for each sample by M1.2 RT-qPCR are shown as black dots (panels A and B); Cq values obtained for each sample by the specific RT-qPCRs are depicted as follows: Panel A red lozenges – HPAI H5, green crosses - LPAI H5; panel B blue lozenges - clade 2.2.1.2, purple triangles - clade 2.3.2.1, ochre Xs - clade 2.3.4.4.; panel C compares categorised Cq values obtained for all samples by M1.2 RT-qPCR (black box-and-whiskers) and the specific RT-qPCRs (colours as described for panels A and B).

**Table 3:** Pathotyping and phylotyping of different potentially zoonotic HPAI and LPAI influenza A subtype H5 virus isolates and field samples collected from poultry and wild bird species in different countries, 2013–2016

No.	Sample ID	Type of sample	Accession Number ^a^	Clade	PCR results					
					M1.2	HPAI H5	LPAI H5	2.2.1.2	2.3.2.1	2.3.4.4
1	A/chicken/Egypt/NLQP33SD-AR748/2013	Isolate	EPI557178	HP 2.2.1.2	27.25	30.72	Neg	27.43	Neg	Neg
2	A/chicken/Egypt/NLQP2AL-AR749/2013	Isolate	EPI557186	HP 2.2.1.2	27.65	30.53	35.56	27.94	Neg	Neg
3	A/duck/Egypt/NLQP27SG-AR750/2013	Isolate	EPI557194	HP 2.2.1.2	28.01	30.01	Neg	27.41	Neg	Neg
4	A/chicken/Egypt/NLQP639V-AR752/2013	Isolate	EPI557202	HP 2.2.1.2	24.11	29.58	Neg	23.32	Neg	Neg
5	A/chicken/Egypt/NLQP20SL-AR751/2013	Isolate	EPI557210	HP 2.2.1.2	28.90	32.90	Neg	29.30	Neg	Neg
6	A/chicken/Egypt/NLQP139V-AR753/2013	Isolate	EPI557218	HP 2.2.1.2	33.32	34.13	Neg	33.51	Neg	Neg
7	A/quail/Egypt/BSU5514-AR2219/2014	Field sample	EPI557138	HP 2.2.1.2	15.12	22.12	Neg	15.47	Neg	Neg
8	A/chicken/Egypt/AR234-FAOF8NLQP/2014	Field sample	EPI573250	HP 2.2.1.2	28.60	31.95	Neg	28.75	Neg	Neg
9	A/turkey/Egypt/AR235-S240NLQP/2014	Field sample	EPI573252	HP 2.2.1.2	22.56	27.94	35.77	22.16	Neg	Neg
10	A/chicken/Egypt/AR3690A/2016	Field sample	SA^b^	HP 2.2.1.2	18.01	18.97	35.29	18.61	Neg	Neg
11	A/chicken/Egypt/AR3706/2016	Field sample	SA^b^	HP 2.2.1.2	19.27	22.98	Neg	20.13	Neg	Neg
12	A/chicken/Egypt/AR3707/2016	Field sample	SA^b^	HP 2.2.1.2	23.39	27.66	Neg	23.71	Neg	Neg
13	A/chicken/Egypt/AR3737/2016	Field sample	SA^b^	HP 2.2.1.2	19.53	24.16	Neg	20.58	Neg	Neg
14	A/chicken/Egypt/AR3741/2016	Field sample	SA^b^	HP 2.2.1.2	20.25	23.08	35.04	21.71	Neg	Neg
15	A/chicken/Egypt/AR3753/2016	Field sample	SA^b^	HP 2.2.1.2	21.22	26.21	35.55	23.10	Neg	Neg
16	A/seagull/Dubai/AR3443–2504.1/2014	Isolate	EPI603554	HP 2.3.2.1	15.62	16.32	Neg	Neg	15.72	Neg
17	A/stone curlew/Dubai/AR3444–2504.2/2014	Isolate	EPI603569	HP 2.3.2.1	13.81	14.72	Neg	Neg	14.70	Neg
18	A/duck/Ivory_Coast/15VIR2742–1/2015	Spleen and caecum	NA	HP 2.3.2.1	31.79	23.56	Neg	Neg	23.93	Neg
19	A/chicken/Ghana/15VIR2588–4/2015	Spleen	KU97137	HP 2.3.2.1	22.72	24.47	Neg	Neg	18.07	Neg
20	A/chicken/Ghana/15VIR2588–10/2015	Cloacal swab	KU971357	HP 2.3.2.1	26.24	26.80	Neg	Neg	24.61	Neg
21	A/chicken/Niger/15VIR2060–12/2015	Tracheal swab	KU971309	HP 2.3.2.1	25.50	25.08	Neg	Neg	24.37	Neg
22	A/chicken/Niger/15VIR2060–5/2015	Swab	KU971326	HP 2.3.2.1	23.08	21.99	Neg	Neg	20.35	Neg
23	A/domestic_bird/Burkina_Faso/15VIR1774–24/2015	Swab	KU971508	HP 2.3.2.1	21.05	29.03	Neg	Neg	24.01	Neg
24	A/domestic_bird/Burkina_Faso/15VIR1774–23/2015	Organ	KU971500	HP 2.3.2.1	21.91	30.83	Neg	Neg	24.72	Neg
25	A/chicken/Ghana/16VIR-4304–1/2016	Organ	SA^b^	HP 2.3.2.1	23.37	22.49	Neg	Neg	18.44	Neg
26	A/chicken/Ghana/16VIR-4304–25/2016	Organ	SA^b^	HP 2.3.2.1	15.51	15.62	Neg	Neg	12.09	Neg
27	A/chicken/Ghana/16VIR-4304–42/2016	Organ	SA^b^	HP 2.3.2.1	24.22	20.45	Neg	Neg	18.28	Neg
28	A/chicken/Ghana/16VIR-4304–9/2016	Organ	SA^b^	HP 2.3.2.1	21.79	21.90	Neg	Neg	19.13	Neg
29	A/duck/Cameroon/16VIR-3791–21/2016	Lung and trachea	SA^b^	HP 2.3.2.1	23.00	18.14	Neg	Neg	17.98	Neg
30	A/chicken/Iraq/AR5282/2016	Field sample	NA	HP 2.3.2.1	28.78	28.20	Neg	Neg	29.57	Neg
31	A/chicken/ Iraq/AR5283/2016	Field sample	NA	HP 2.3.2.1	31.70	31.12	Neg	Neg	35.02	Neg
32	A/chicken/Iraq/AR5286/2016	Field sample	SA^b^	HP 2.3.2.1	28.21	28.10	36.50	Neg	28.16	Neg
33	A/chicken/Iraq/AR5287/2016	Field sample	SA^b^	HP 2.3.2.1	28.05	27.08	Neg	Neg	26.80	Neg
34	A/chicken/Iraq/AR5291/2016	Field sample	SA^b^	HP 2.3.2.1	29.29	29.09	Neg	Neg	28.09	Neg
35	A/chicken/Iraq/AR5292/2016	Field sample	NA	HP 2.3.2.1	30.83	30.28	36.32	Neg	31.15	Neg
36	A/chicken/Iraq/AR5296/2016	Field sample	SA^b^	HP 2.3.2.1	28.60	28.21	Neg	Neg	28.53	Neg
37	A/turkey/Germany/AR2499/2014	Field sample	SA^b^	HP 2.3.4.4	27.78	26.48	36.71	Neg	Neg	24.61
38	A/turkey/Germany/AR2500/2014	Field sample	SA^b^	HP 2.3.4.4	29.59	27.44	Neg	Neg	Neg	25.20
39	A/turkey/Germany/AR2501/2014	Field sample	SA^b^	HP 2.3.4.4	32.21	30.65	Neg	Neg	Neg	28.30
40	A/turkey/Germany/AR2502/2014	Field sample	SA^b^	HP 2.3.4.4	30.08	27.92	Neg	Neg	Neg	25.67
41	A/turkey/Germany/AR2503/2014	Field sample	SA^b^	HP 2.3.4.4	30.52	28.21	Neg	Neg	Neg	25.92
42	A/turkey/Germany/AR2562/2014	Field sample	SA^b^	HP 2.3.4.4	26.15	25.02	36.88	Neg	Neg	25.21
43	A/turkey/Germany/AR2574/2014	Field sample	SA^b^	HP 2.3.4.4	27.49	30.23	36.36	Neg	Neg	28.01
44	A/turkey/Germany/AR2591/2014	Field sample	SA^b^	HP 2.3.4.4	28.09	30.06	37.13	Neg	Neg	28.57
45	A/teal/Germany/AR2917/2014	Field sample	SA^b^	HP 2.3.4.4	31.60	30.08	36.82	Neg	Neg	35.41
46	A/turkey/Germany/AR3372/2014	Field sample	EPI553172	HP 2.3.4.4	26.33	24.85	Neg	Neg	Neg	23.07
47	A/turkey/Germany/AR3376/2014	Field sample	SA^b^	HP 2.3.4.4	26.39	25.10	Neg	Neg	Neg	23.12
48	A/turkey/Germany/AR3381/2014	Field sample	SA^b^	HP 2.3.4.4	26.85	25.26	Neg	Neg	Neg	23.40
49	A/turkey/Germany/AR3382/2014	Field sample	SA^b^	HP 2.3.4.4	27.64	26.18	Neg	Neg	Neg	24.18
50	A/turkey/Germany/AR3383/2014	Field sample	SA^b^	HP 2.3.4.4	29.26	28.13	Neg	Neg	Neg	26.06
51	A/duck/Germany/AR3457/2014	Field sample	SA^b^	HP 2.3.4.4	30.29	29.30	Neg	Neg	Neg	28.34
52	A/duck/Germany/AR3465/2014	Field sample	SA^b^	HP 2.3.4.4	23.98	23.15	Neg	Neg	Neg	20.70
53	A/duck/Germany/AR3470/2014	Field sample	SA^b^	HP 2.3.4.4	22.15	23.89	Neg	Neg	Neg	21.78
54	A/wild-duck/Germany/AR8603/2016	Field sample	SA^b^	HP 2.3.4.4b	22.51	22.90	Neg	Neg	Neg	21.14
55	A/greyleg goose /Germany/AR8604/2016	Field sample	SA^b^	HP 2.3.4.4b	22.41	22.30	Neg	Neg	Neg	20.26
56	A/greater scaup/Germany/AR9090/2016	Field sample	SA^b^	HP 2.3.4.4b	27.29	31.71	Neg	Neg	Neg	29.61
57	A/greater scaup/Germany/AR9091/2016	Field sample	SA^b^	HP 2.3.4.4b	28.95	34.40	Neg	Neg	Neg	31.74
58	A/greater scaup/Germany/AR9092/2016	Field sample	SA^b^	HP 2.3.4.4b	23.85	25.70	Neg	Neg	Neg	26.05
59	A/grey heron/Germany/AR9093/2016	Field sample	SA^b^	HP 2.3.4.4b	20.10	22.62	Neg	Neg	Neg	22.44
60	A/greater scaup/Germany/AR9094/2016	Field sample	SA^b^	HP 2.3.4.4b	16.31	20.62	Neg	Neg	Neg	18.92
61	A/greater scaup/Germany/AR9095/2016	Field sample	SA^b^	HP 2.3.4.4b	20.99	22.79	Neg	Neg	Neg	21.75
62	A/northern pintail /Germany/AR9096/2016	Field sample	SA^b^	HP 2.3.4.4b	22.83	28.12	Neg	Neg	Neg	23.95
63	A/bean goose/Germany/AR9097/2016	Field sample	SA^b^	HP 2.3.4.4b	22.97	24.88	Neg	Neg	Neg	24.92
64	A/herring gull /Germany/AR9098/2016	Field sample	SA^b^	HP 2.3.4.4b	20.40	22.35	Neg	Neg	Neg	23.64
65	A/mute swan/Germany/AR9099/2016	Field sample	SA^b^	HP 2.3.4.4b	21.12	25.83	Neg	Neg	Neg	22.75
66	A/chicken/Germany/AR9140/2016	Field sample	SA^b^	HP 2.3.4.4b	21.08	20.55	Neg	Neg	Neg	22.92
67	A/chicken/Germany/AR9141/2016	Field sample	SA^b^	HP 2.3.4.4b	21.21	20.13	Neg	Neg	Neg	23.12
68	A/chicken/Germany/AR9143/2016	Field sample	SA^b^	HP 2.3.4.4b	32.27	31.51	Neg	Neg	Neg	35.49
69	A/chicken/Germany/AR9144/2016	Field sample	SA^b^	HP 2.3.4.4b	21.89	20.89	Neg	Neg	Neg	25.92
70	A/chicken/Italy/22/1998	Isolate	CAP58165	LPAI H5N9	12.34	Neg	19.06	Neg	Neg	Neg
71	A/mallard/Germany/Wv1349–51K/2003	Isolate	CAP58164	LPAI H5N3	26.00	Neg	14.79	Neg	Neg	Neg
72	A/mallard/Germany/Wv476/2004	Isolate	NA	LPAI H5N2	29.87	Neg	29.5	Neg	Neg	Neg
73	A/mallard/Germany/Wv474–77K/2004	Isolate	NA	LPAI H5N2	29.64	Neg	34.81	Neg	Neg	Neg
74	A/ostrich/Germany/R5–10/2006	Isolate	HF563057	LPAI H5N3	26.80	Neg	26.19	Neg	Neg	Neg
75	A/mallard/Germany/R2557/2006	Isolate	NA	LPAI H5N3	26.24	Neg	27.61	Neg	Neg	Neg
76	A/mallard/Germany/R731/2008	Isolate	SA^b^	LPAI H5N3	30.36	Neg	32.30	Neg	Neg	Neg
77	A/mallard/Germany/R771/2008	Isolate	SA^b^	LPAI H5N3	29.24	Neg	30.28	Neg	Neg	Neg
78	A/mallard/Germany/R772/2008	Isolate	SA^b^	LPAI H5N3	23.68	Neg	24.50	Neg	Neg	Neg
79	A/turkey/Germany/R1550/2008	Isolate	NA	LPAI H5N3	25.17	Neg	26.22	Neg	Neg	Neg
80	A/turkey/Germany/R1551/2008	Isolate	NA	LPAI H5N3	24.03	Neg	24.91	Neg	Neg	Neg
81	A/turkey/Germany/R1557/2008	Isolate	SA^b a^	LPAI H5N3	23.50	Neg	24.16	Neg	Neg	Neg
82	A/turkey/Germany/R1612/2008	Isolate	NA	LPAI H5N3	27.43	Neg	29.25	Neg	Neg	Neg
83	A/turkey/Germany/R2014/2008	Isolate	SA^b^	LPAI H5N3	24.33	Neg	21.99	Neg	Neg	Neg
84	A/turkey/Germany/R2015/2008	Isolate	SA^b^	LPAI H5N3	15.69	Neg	30.13	Neg	Neg	Neg
85	A/turkey/Germany/R2016/2008	Isolate	SA^b^	LPAI H5N3	18.13	Neg	15.96	Neg	Neg	Neg
86	A/turkey/Germany/R2017/2008	Isolate	SA^b^	LPAI H5N3	18.32	Neg	16.92	Neg	Neg	Neg
87	A/turkey/Germany/R2018/2008	Isolate	SA^b^	LPAI H5N3	14.16	Neg	16.05	Neg	Neg	Neg
88	A/turkey/Germany/R2019/2008	Isolate	SA^b^	LPAI H5N3	14.55	Neg	16.84	Neg	Neg	Neg
89	A/turkey/Germany/R2020/2008	Isolate	SA^b^	LPAI H5N3	19.38	Neg	16.73	Neg	Neg	Neg
90	A/turkey/Germany/R2021/2008	Isolate	SA^b^	LPAI H5N3	12.71	Neg	13.51	Neg	Neg	Neg
91	A/turkey/Germany/R2022/2008	Isolate	SA^b^	LPAI H5N3	12.63	Neg	13.18	Neg	Neg	Neg
92	A/turkey/Germany/R2023/2008	Isolate	SA^b^	LPAI H5N3	19.37	Neg	17.07	Neg	Neg	Neg
93	A/turkey/Germany/R2024/2008	Isolate	SA^b^	LPAI H5N3	22.52	Neg	20.39	Neg	Neg	Neg
94	A/turkey/Germany/R2025/2008	Isolate	SA^b^	LPAI H5N3	22.44	Neg	25.22	Neg	Neg	Neg
95	A/turkey/Germany/R2026/2008	Isolate	SA^b^	LPAI H5N3	14.70	Neg	16.26	Neg	Neg	Neg
96	A/turkey/Germany/R2027/2008	Isolate	SA^b^	LPAI H5N3	17.80	Neg	16.06	Neg	Neg	Neg
97	A/mallard/Germany/R2892–94/2009	Isolate	EPI356412	LPAI H5N3	11.98	Neg	14.37	Neg	Neg	Neg
98	A/duck/Germany/AR1965/2013	Field sample	NA	LPAI H5N3	26.62	Neg	27.25	Neg	Neg	Neg
99	A/turkey/Germany/AR1892/1/2014	Field sample	SA^b^	LPAI H5N2	20.03	Neg	21.15	Neg	Neg	Neg
100	A/duck/Germany/AR1/2015	Field sample	SA^b^	LPAI H5N3	29.20	Neg	34.01	Neg	Neg	Neg
101	A/swan/Germany/AR111/2015	Field sample	SA^b^	LPAI H5N4	27.45	Neg	31.02	Neg	Neg	Neg
102	A/goose/Germany/AR398/2015	Field sample	SA^b^	LPAI	31.09	Neg	33.69	Neg	Neg	Neg
103	A/duck/Germany/AR1231/1/2015	Field sample	NA	LPAI H5N2	26.74	Neg	32.91	Neg	Neg	Neg
104	A/duck/Germany/AR2853/15–1/2015	Field sample	SA^b^	LPAI H5N3	27.06	Neg	26.25	Neg	Neg	Neg
105	A/goose/Germany/AR3264/1/2015	Field sample	SA^b^	LPAI H5N2	34.47	Neg	35.50	Neg	Neg	Neg
106	A/wild bird/Germany/AR221/2015	Field sample	SA^b^	LP H5N3	22.17	Neg	23.48	Neg	Neg	Neg

HA: haemagglutinin; HP: highly pathogenic; HPAI: highly pathogenic avian influenza; ID: identity; LP: low pathogenic; LPAI: low pathogenic avian influenza; NA: sequence not available; Neg: negative; SA: sequence available.

^a^ Sequences were obtained from GenBank at the National Center for Biotechnology Information (NCBI) or the EpiFlu database of the Global Initiative on Sharing Avian Influenza Data (GISAID).

^b^ Sequenced in the frame of the current study; sequences available from the authors upon request.

All pathotyping results matched the results obtained by nt sequence analysis of the HA cleavage site. However, in a few samples (two isolates, 10 clinical samples) of HP viruses, the LPAI H5 RT-qPCR also gave a weak positive signal (Cq > 35). Compared with the LPAI H5 signal the HPAI H5 signal of these samples yielded Cq values 6–10 units lower on average ascertaining good diagnostic specificity. Depending on the clade, the HP phenotype was detected with equal (clade 2.3.2.1) or slightly reduced (clade 2.2.1.2) sensitivity; the LP H5 RT-qPCR appeared to be slightly less sensitive than the M PCR as far as clinical samples were concerned (Table 3; Figure 2a and c). Sequences across the cleavage sites of these samples are presented in a supplemental alignment (Figure 2).

In a next step, the samples that were designated HPAI H5-positive were subjected to the three phylotyping RT-qPCRs. Here, 15, 21 and 33 samples, respectively, were exclusively positive for either clade 2.2.1.2, 2.3.2.1 or 2.3.4.4 (Table 3). Thus, a clear cut clade assignment was possible for all gs/GD HP H5 samples. Results were counterchecked by feeding available HA sequences of these samples into the IRD clade prediction tool (www.fludb.org/brc/h5n1-Classifier.spg?method=ShowCleanInputPage&decorator=influenz): In all cases the same clade was assigned by sequence analysis and by PCR. In a final step also all LPAI H5 samples were tested in the phylotyping RT-qPCRs and none of them cross-reacted. Regarding the sensitivity of these PCRs, the Cq values were compared with those of the generic M1.2-specific RT-qPCR (Figure 2b). For clade 2.2.1.2 and 2.3.2.1 the sensitivity was almost identical to the M PCR; for clade 2.3.4.4a, the clade-specific PCR proved to be slightly more sensitive while viruses of clade 2.3.4.4b were detected at a slightly lower sensitivity; detection of clade 2.3.4.4b viruses was slightly less sensitive than the M PCR (Figure 2b and c; Table 3) as far as clinical samples were concerned.

Rank Sum tests implemented in the SigmaPlot software package were performed and no statistically significant difference between the median Cq values of each specific assay and the M1.2 RT-qPCR assay was found (p > 0,50) indicating that the newly developed RT-qPCRs display similar analytical sensitivity. Thus, the phylotyping RT-qPCRs allow a sensitive and highly specific detection and distinction of the three major gs/GD clades currently circulating in countries where the viruses were obtained from.

## Discussion

Rapid molecular diagnosis including patho- and phylotyping is basis to enable measures aimed at repressing the spread of potentially zoonotic HPAI viruses. The TaqMan PCR technology has proven reliable, versatile, and comparatively cost-effective in the generic detection and subtype differentiation of AIV [30]. Further differentiation of clades, lineages and pathotypes was previously nearly entirely based on nt sequencing approaches which require expensive equipment and are time consuming. In epidemiologically complex settings where different lineages and pathotypes of potentially zoonotic and notifiable infectious agents co-circulate, a more rapid and direct access to testing and results, e.g. by using RT-qPCRs, is desirable. Although RT-qPCRs are inferior to sequencing techniques in terms of retrievable data details, they are superior with respect to time-to-diagnosis and ease-of-use. This concept which we used earlier for pathotyping of H5N1 [31], was here further extended and refined for the identification and discrimination of avian influenza A subtype H5 viruses of different patho- and phylotypes. The focus was put on those clades of H5 viruses (2.2.1.2, 2.3.2.1, 2.3.4.4) that had previously ‘escaped’ from Asia and were detected in western parts of Eurasia and in Africa.

Pathotyping of avian influenza A subtype H5 viruses is mandatory from an animal health perspective. The pathotyping RT-qPCRs presented here reduce time-to-diagnosis to just three hours following sample receipt. To our knowledge this is the broadest and most detailed attempt of AIV pathotyping using RT-qPCR. The availability of highly sensitive pathotyping PCRs would also allow to detect mixtures of HP and LP H5 viruses in the same sample; in fact, some of our HP-positive field samples also gave weak LP signals (Table 3, sample numbers 2, 9, 10, 14, 15, 30, 32, 35, 37, 42–5). Yet, LPAI pathotypes in these samples were detected at distinctly higher Cq values indicating either a minor population in a quasispecies of different pathotypes or expressing some cross-reactivity of LPAI primers and probe; in any case, the detection of HPAI genotypes as a major population in a set of field samples was always unequivocal. Further insight into the true nature of these mixtures would only be unravelled by deep sequencing approaches of those samples.

Rapid pathotyping enables rapid implementation of appropriate measures to prevent further spread of virus such as closure of poultry holdings and/or live poultry markets, culling of infected flocks etc. This impedes accumulation of potentially zoonotic AIV at the poultry-human interface which in turn lowers the risks of human infection.

Phylotyping of gs/GD HPAI H5 virus clades is important since each clade, and often also sublineages thereof, display distinct antigenic and pathogenetic properties. This has direct implications, as by assigning the matching clade, appropriate vaccines that ensure the closest antigenic match with the circulating viruses can be selected [32,33]. In particular, countries where gs/GD viruses have become endemic in poultry populations, rely on vaccination of poultry on a broad scale to suppress circulating viruses and to limit risks of human exposure [23]. However, it should be noted that mutant escape variants within these clades selected by vaccine-induced population immunity will not be detected as such by the assays, and in fact, such mutants may also be detected at lower sensitivity if primer and/or probe binding sites are affected by mutations. Detection of variants will still depend on either nt sequencing or virus isolation/antigenic characterisation approaches but the newly developed assays will aid in selection of meaningful samples in this respect. In particular, samples that do not give conclusively similar Cq values in the generic and the specific assays should prompt in-depth analysis by nt sequencing.

It should be clearly stated that the assays presented here have limitations owed to the restricted geographical distribution of the targeted clades. The use of the newly developed PCRs in regions where viruses belonging to the targeted clades (2.2.1.2, 2.3.2.1c and 2.3.4.4) are reportedly absent is only recommended if immediate incursions with any of these clades are apprehended. Phylotyping indirectly may point towards zoonotic potential since different gs/GD lineages vary in their zoonotic propensity: Egyptian 2.2.1.2 viruses are characterised by increased affinity to human-like sialic acid receptors and have caused by far the largest number of human influenza A(H5N1) virus infections over the past decade [12]. For clade 2.3.2.1c viruses, repeatedly detected in the Middle East (excluding Egypt) and endemic in Western African countries, only few human cases have been recorded. The 2.3.4.4 viruses currently present in various parts of Europe have not provoked human infection so far [34].

Extended co-circulation of more than one gs/GD lineage in poultry and/or wild birds in a wider geographic region was repeatedly reported [35,36]. It is pivotal, for the above mentioned reasons, to detect incursions of distinct HPAI virus lineages in a timely manner. In this respect, the newly developed RT-qPCR assays were shown to be useful tools for an improved rapid and simple characterisation of patho- and phylotypes of Eurasian origin avian influenza A subtype H5 viruses. The assays aid in speeding up diagnosis on clinical samples because the time consuming (initial) need of virus isolation and nt sequencing is avoided. Given the high substitution rate of HP H5 influenza viruses frequent checks and, if required, updates of the primers and probes are recommended to ensure full specificity and sensitivity of the patho- and phylotyping RT-qPCRs. These PCRs are advantageous in particular for wild bird samples, especially those that contain LPAI viruses, often with low viral loads and therefore fail to yield replication-competent virus. With respect to HPAI virus, the renouncement from initial virus isolation improves biosecurity. However, the presented assays are not intended to replace virus isolation and antigenic characterisation as a means to detect emerging antigenic drift mutants. Nevertheless, they may aid in selection of appropriate samples for such tasks. Accurate phylotyping also facilitates selection of appropriate vaccines as it serves as an early warning for the incursion of new and antigenically possibly distinct phylotypes.

**Conclusions**

The assays reported here are primarily intended for screening purposes of avian samples; confirmatory assays, including nt sequence analyses and antigenic characterisation, are still required for new incursions and outbreak scenarios that feature an expansion of the geographic area and/or the range of affected species or poultry sectors. When used in the frame of on-going outbreaks, in particular in regions where vaccination is not used as a preventive measure, results of the patho- and phylotyping PCRs are deemed solid enough for reporting purposes and to justify the implementation of restriction measures. In such settings, similar to the current outbreaks of clade 2.3.4.4b HP H5N8 in Europe, the assays can be prioritised to running the HP and only one (i.e. the fitting) of the phylotyping PCRs on M1.2- and H5 PCR-positive samples. This significantly speeds up time-to-diagnosis and reduces reaction times in a OneHealth approach of repressing the spread of gs/GD HP AIV. Sequencing facilities, classically required for patho- and phylotyping, may not be available, and even not logistically accessible in many regions severely affected by H5 HPAI incursions. The prospect of having sequencing-independent, TaqMan-based specific and sensitive typing assays, as described here, available in developing regions is expected to boost regional diagnostic capacities eventually leading to improved disease control.
